# Multi-scale structural and chemical analysis of sugarcane bagasse in the process of sequential acid–base pretreatment and ethanol production by *Scheffersomyces shehatae* and *Saccharomyces cerevisiae*

**DOI:** 10.1186/1754-6834-7-63

**Published:** 2014-04-16

**Authors:** Anuj K Chandel, Felipe AF Antunes, Virgilio Anjos, Maria JV Bell, Leonarde N Rodrigues, Igor Polikarpov, Eduardo R de Azevedo, Oigres D Bernardinelli, Carlos A Rosa, Fernando C Pagnocca, Silvio S da Silva

**Affiliations:** 1 Department of Biotechnology, School of Engineering of Lorena, Estrada Municipal do Campinho, University of São Paulo, Caixa Postal 116 12.602.810, Lorena, São Paulo, Brazil; 2 Materials Spectroscopy Laboratory, Department of Physics, Federal University of Juiz de Fora, Juiz de Fora 36036-330 Minas Gerais, Brazil; 3 Instituto de Física de São Carlos, Universidade de São Paulo, Caixa Postal 369, São Carlos, São Paulo CEP 13560-970, Brazil; 4 Departmento de Microbiologia, Instituto de Ciências Biológicas, Universidade Federal de Minas Gerais, Belo Horizonte, Minas Gerais, Brazil; 5 Department of Biochemistry and Microbiology, Institute of Biosciences, CIES/UNESP, Rio Claro, São Paulo, Brazil

**Keywords:** Sugarcane bagasse, Sequential acid–base pretreatment, Enzymatic hydrolysis, Structural analysis, Bioethanol, Yeasts

## Abstract

**Background:**

Heavy usage of gasoline, burgeoning fuel prices, and environmental issues have paved the way for the exploration of cellulosic ethanol. Cellulosic ethanol production technologies are emerging and require continued technological advancements. One of the most challenging issues is the pretreatment of lignocellulosic biomass for the desired sugars yields after enzymatic hydrolysis. We hypothesized that consecutive dilute sulfuric acid-dilute sodium hydroxide pretreatment would overcome the native recalcitrance of sugarcane bagasse (SB) by enhancing cellulase accessibility of the embedded cellulosic microfibrils.

**Results:**

SB hemicellulosic hydrolysate after concentration by vacuum evaporation and detoxification showed 30.89 g/l xylose along with other products (0.32 g/l glucose, 2.31 g/l arabinose, and 1.26 g/l acetic acid). The recovered cellulignin was subsequently delignified by sodium hydroxide mediated pretreatment. The acid–base pretreated material released 48.50 g/l total reducing sugars (0.91 g sugars/g cellulose amount in SB) after enzymatic hydrolysis. Ultra-structural mapping of acid–base pretreated and enzyme hydrolyzed SB by microscopic analysis (scanning electron microcopy (SEM), transmitted light microscopy (TLM), and spectroscopic analysis (X-ray diffraction (XRD), Fourier transform infrared (FTIR) spectroscopy, Fourier transform near-infrared (FT-NIR) spectroscopy, and nuclear magnetic resonance (NMR) spectroscopy) elucidated the molecular changes in hemicellulose, cellulose, and lignin components of bagasse. The detoxified hemicellulosic hydrolysate was fermented by *Scheffersomyces shehatae* (syn. *Candida shehatae* UFMG HM 52.2) and resulted in 9.11 g/l ethanol production (yield 0.38 g/g) after 48 hours of fermentation. Enzymatic hydrolysate when fermented by *Saccharomyces cerevisiae* 174 revealed 8.13 g/l ethanol (yield 0.22 g/g) after 72 hours of fermentation.

**Conclusions:**

Multi-scale structural studies of SB after sequential acid–base pretreatment and enzymatic hydrolysis showed marked changes in hemicellulose and lignin removal at molecular level. The cellulosic material showed high saccharification efficiency after enzymatic hydrolysis. Hemicellulosic and cellulosic hydrolysates revealed moderate ethanol production by *S. shehatae* and *S. cerevisiae* under batch fermentation conditions.

## Background

Harnessing the carbohydrates from lignocellulosic biomass into bioethanol is not only a ‘nice idea’ but an ‘important necessity’ owing to the increased energy demand globally, safe environment, and sustainable employment [1]. Among the most feasible second generation feedstock for bioethanol production, sugarcane bagasse (SB), a fibrous residue generated during the extraction of cane juice in mills, is an excellent raw commodity due to large abundance, non-competitiveness with food or feed requirement, easy transportation, and rich in accessible carbohydrates [2]. The fullest exploitation of SB in the integrated biorefineries setting (1G + 2G ethanol production in one unit) may provide a unique breakthrough for the commercialization of biofuels [3].

The key driver for the successful conversion of SB into ethanol is the selection of efficient pretreatment technology followed by maximal sugars recovery coupled with ethanol production with desired yield and productivities [2,4,5]. Pretreatment of lignocellulose is an inevitable process for the commercial deployment of cellulosic biofuels. Nevertheless, there are only a few robust pretreatment technologies available which efficiently enable the cellulosic fraction of the cell wall for enzymatic conversion into monomeric sugars [5,6]. Sequential acid–base pretreatment has shown efficient removal of hemicellulose and lignin from SB eventually releasing high amounts of fermentable sugars upon enzymatic hydrolysis [7-9]. Apart from acid–base pretreatments, several pretreatment methods have been studied in the past which selectively remove either hemicellulose or lignin from the SB matrix. The choice of effective pretreatment methods depends upon the minimum degradation of carbohydrate, ability to enhance the surface area of cellulosic substrates, and minimizing the production of inhibitors and toxic products [1,5].

SB is consisted of crystalline cellulose nanofibrils embedded in an amorphous matrix of cross-linked lignin and hemicelluloses that impair enzyme and microbial accessibility [10]. Structural changes in the cellular components of SB elucidating the hemicellulose degradation and removal of lignin after sequential acid–base pretreatment are very important for the investigation of changes in the cell wall at molecular level [7]. Structural mapping of lignocellulosic materials after various thermochemical pretreatments provide important insights at molecular level to facilitate the mechanistic action of catalysts used for pretreatment [10-13]. Microbial conversion of hemicellulose and cellulose derived sugars into ethanol with optimum yields and productivities are the key parameters to obtain cheap ethanol in biorefineries [2]. For the conversion of xylose, *Scheffersomyces shehatae* (syn. *Candida shehatae*) is the preferred choice [14,15]. On the other hand, *Saccharomyces cerevisiae* is the most employed microorganism for ethanol production from cellulosic derived sugars with high productivities [16].

In the present study, we attempted to pretreat the SB by sequential acid–base followed by enzymatic saccharification using commercial enzymes. The hemicellulosic hydrolysate after vacuum concentration and detoxification (30.89 g/l xylose) and cellulosic hydrolysate (48.50 g/l glucose) were fermented by *S. shehatae* UFMG HM 52.2 and *S. cerevisiae* 174 to ethanol under batch cultivation conditions. Further, efforts were made to unveil the structure of native, sulfuric acid pretreated, sodium hydroxide delignified, and enzyme-digested SB by using scanning electron microscopy (SEM), transmitted light microscopy (TLM), Fourier transform infrared (FTIR) spectroscopy, Fourier transform near-infrared (FT-NIR) spectroscopy, micro-Raman spectroscopy, and solid-state nuclear magnetic resonance (NMR) spectroscopy.

## Results and discussion

Native SB used in this study was found to contain cellulose (39.52%), hemicellulose (25.63%), total lignin (30.36%), ash (1.44%), and extractives (2.90%). These values are within the range found in other studies. The composition of SB varies with variety, origin, cultivation type of sugarcane, and the analytical method used for the characterization [5]. For instance, Rocha *et al*. [17] observed 45.5% cellulose, 27% hemicellulose, 21.1% lignin, and 2.2% ash in SB. Rabelo *et al*. [18] observed 38.4% cellulose, 23.2% hemicellulose, 25% lignin, and 1.5% ash in SB.

### Dilute sulfuric acid treatment

Dilute sulfuric acid pretreatment of lignocellulosics is a fast and effective process for the removal of hemicellulose, leaving cellulose and lignin together in compact form, the so-called cellulignin [5]. The hemicellulose fraction of the plant cell wall depolymerizes into its monomeric sugar constituents of primarily xylose in addition to other cell wall derived compounds. There are several compounds present in the dilute acid hydrolysate of SB. Examples of commonly found compounds are aliphatic acids (acetic, formic, and levulinic acid), furan derivatives (furfural and hydroxymethylfurfural (HMF)), and phenolic compounds. Table 1 shows the composition of dilute acid hydrolysate. The generation of these compounds depends upon the chemical composition of the cell wall and the conditions employed during dilute acid hydrolysis [5,19].

**Table 1:** Chemical composition of sugarcane bagasse (SB) native hemicellulose hydrolysate, vacuum concentrated, and detoxified (overliming plus activated charcoal)

**Compound (g/l)**	**Crude hydrolysate**	**Concentrated hydrolysate (3x)**	**Detoxified hydrolysate**
Glucose	nd	0.39	0.31
Xylose	10.9	37.44	30.89
Arabinose	nd	2.73	2.31
Acetic acid	0.53	1.83	1.26
HMF	0.005	6 × 10^-3^	5.93 × 10^-4^
Furfural	0.02	2.85 × 10^-3^	1 × 10^-3^
pH	1.0	0.5	5.5

HMF, hydroxymethylfurfural; nd, not detectable; SB, sugarcane bagasse.

After the dilute acid hydrolysis, 92.78% hemicellulose was removed, which in turn increased the amount of lignin (45.09%), cellulose (48.95%), and ash (3.2%) in the substrate, showing the efficiency of this pretreatment. Extractives were found in negligible amounts. Canilha *et al*. [20] reported 3.7% hemicellulose in the substrate after the dilute sulfuric acid pretreatment (2.5% w/v H_2_SO_4_, 150°C, 30 minutes) from the dilute sulfuric acid pretreated SB. Rezende *et al*. [7] recovered 51.2% cellulose, 29.5% lignin, and 7.8% hemicellulose in the dilute sulfuric acid pretreated SB (1% H_2_SO_4_, 120°C, 40 minutes).

### Vacuum evaporation and detoxification of dilute acid hydrolysate

In order to increase the concentration of sugars in the hydrolysate, native hemicellulosic hydrolysate was concentrated by vacuum evaporation at 70°C. The hydrolysate after concentration was found to contain an efficient amount of sugars (37.44 g/l xylose, 2.73 g/l arabinose, and 0.39 g/l glucose) and other undesired products (Table 1). It is essential to eliminate these inhibitors from the hydrolysate before fermentation to obtain the desired ethanol yield and productivities [19]. Sequential detoxification strategy (CaO mediated overliming followed by activated charcoal treatment) has shown an effective removal of inhibitors from the hydrolysates without the loss of sugars (reduction of approximately 96% of furfural and 90% of HMF comparing crude with treated hydrolysate). However, the removal of acetic acid was negligible (Table 1). Activated charcoal treatment effectively eliminates the furfurals and HMF from dilute acid hydrolysate of SB under the process conditions employed in this study. These results are in good accordance with the previous study on detoxification of SB acid hydrolysate by overliming and charcoal [19].

### Sodium hydroxide treatment

The recovered cellulignin after dilute acid hydrolysis was delignified by sodium hydroxide pretreatment (1.0% w/v NaOH, 120°C, 60 minutes). These conditions showed the maximal removal of lignin (55.65%) from the substrate after a response surface optimization (L9 Taguchi orthogonal design of experiments) for delignification [9]. It is necessary to remove the lignin from the substrate to obtain the desired sugars recovery from cellulignin after enzymatic saccharification. Lignin significantly aids the biomass recalcitrance eventually strongly impairing the cellulase amenability towards the substrate.

Sodium hydroxide mediated pretreatment disrupts the SB cell wall by solubilization of the remaining hemicellulose and lignin. Sodium hydroxide mechanistically cleavages the alpha-aryl ester bonds from its polyphenolic monomers along with weakening of hydrogen bonds, which in turn promotes the swelling of cellulose [21]. Sequential acid–base pretreatment of SB has been found effective for the removal of hemicellulose and lignin from SB increasing the amenability of cellulases towards cellulose [7,8]. NaOH mediated delignification of cellulignin (1% w/v NaOH, 120°C, 1 hour of pretreatment time) showed 76.5% cellulose, 20.0% lignin, and 3.50% ash in the substrate. Rezende *et al*. [7] reported 88% lignin removal which showed 84.7% cellulose and 3.3% hemicellulose in acid-alkali pretreated SB (2% w/v NaOH, 120°C, 40 minutes) [7]. Recently, Rocha *et al*. [21] observed 91.2% lignin removal from cellulignin of SB under the alkali pretreatment conditions (1% w/v NaOH, 100°C, 1 hour).

### Enzymatic hydrolysis

The acid–base pretreated SB, the co-called SB-cellulose, was enzymatically saccharified to obtain clean sugar stream (glucose solution). A maximum of 48.50 g/l glucose (0.91 g/g SB-cellulose) was obtained from the acid–base pretreated SB after 96 hours of enzymatic hydrolysis (15 FPU/g, 20 CBU/g of enzyme loading). Acid pretreated bagasse (cellulignin) showed only 22.75 g/l sugars recovery proving the requirement of alkali mediated delignification.

Apart from the efficient pretreatment of the substrate, appropriate enzyme loading and hydrolytic conditions are necessary to obtain the maximum sugars recovery from the substrate [7,22]. Sequential acid–base pretreatment efficiently removes the hemicellulose and lignin from the substrate simultaneously promoting the swelling of cellulose. Further, Tween 20 was also added in the hydrolytic reaction as surfactant for the enhancement of specific surface area for better enzyme action. Surfactants generally enhance the surface area of lignocellulosic substrates to improve the extent of enzymatic hydrolysis. Non-ionic surfactant-like Tween 20 is more effective due to its adsorption on hydrophobic surfaces mainly composed of lignin fragments [22].

It is difficult to compare the hydrolytic efficiency of acid–base pretreated SB with the existing reports due to the different conditions employed in the studies. For example, Giese *et al*. [8] performed sequential acid–base pretreatment of SB (100 mg H_2_SO_4_/g of SB, 121°C, 20 minutes) plus alkali (1% w/v NaOH, 100°C, 1 hour). The acid–base pretreated SB was further enzymatically hydrolyzed (cellulase AF28918 300 IU/ml, cellobiase 80 IU/ml, Tween 80 0.15 g/g, and pretreated SB 5% (w/v)), which led to sugars recovery (0.89 g total reducing sugars (TRS)/g). Rezende *et al*. [7] reported 72% cellulose conversion from consecutive acid (1% v/v H_2_SO_4_, 120°C, 40 minutes) and base (1% w/v NaOH, 120°C, 40 minutes) pretreated SB after enzymatic hydrolysis (25 FPU of Accelerase 1500 (DuPont, Wilmington, DE, USA) and 50 IU of β-glucanase from Novozym 188 (Sigma Aldrich, USA).

### Structural mapping

#### Scanning electron microscopy (SEM)

**Figure 1:**
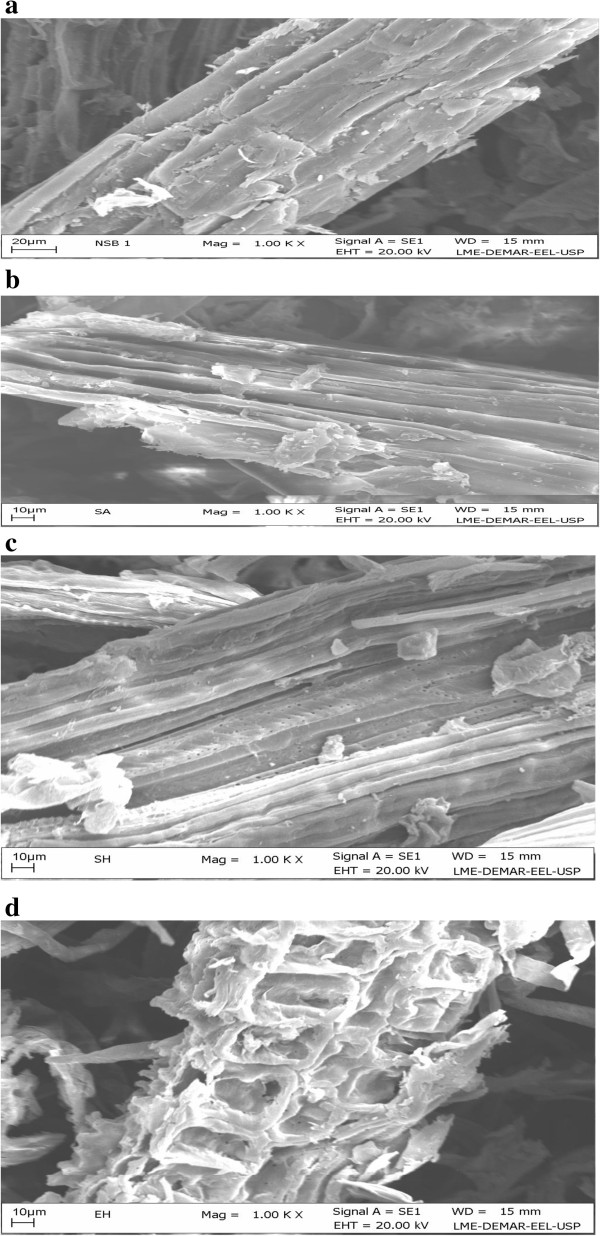
**Scanning electron microscopic (SEM) analysis of sugarcane bagasse (SB).** Showing surface image of **(a)** natural, **(b)** dilute sulfuric acid pretreated, **(c)** sodium hydroxide pretreated cellulignin, and **(d)** enzyme hydrolyzed bagasse. SB, sugarcane bagasse; SEM, scanning electron microscopy.

The ultra-structure of the native SB by SEM was compared with the dilute sulfuric acid pretreated SB (cellulignin), alkali pretreated cellulignin, and enzymatically hydrolyzed substrate. Figure 1a shows the anatomy of milled SB (native) which is compact, rough, and has thick-walled fiber cells interlinked with pith. Fibers are constituted by parallel stripes and are superficially covered with extractives. The most apparent effect of sulfuric acid pretreatment is the separation of fibers from pith and loosening of the fibrous network. Dilute acid pretreatment removes hemicellulose from bagasse disrupting the cell wall with a loose matrix (Figure 1b). However, there were no pores visible. After the sodium hydroxide pretreatment of cellulignin, lignin was removed substantially and the surface of the substrate was very smooth with the appearance of parallel sheaths. Appearance of pores and lignin droplets can also be seen in NaOH pretreated SB (Figure 1c). Rezende *et al*. [7] also observed similar morphological changes in the bagasse surface after sequential acid–base pretreatment [7]. They observed considerable removal of the pith from fibers during dilute sulfuric acid pretreatment followed by extensive dismantling of vascular bundles and detachment of fibers in NaOH pretreated cellulignin. Similarly, Martin *et al*. [23] found the heterogeneous nature of the cell wall, showing the rough cell wall surface after dilute sulfuric acid pretreatment and unpacked fibers with an open structure and smooth surface of bagasse. Enzymatic hydrolysis of acid–base pretreated substrate completely destructed the cellulose, which in turn showed the unstructured and heterogeneous nature of samples (Figure 1d).

### Transmitted light microscopy (TLM)

**Figure 2:**
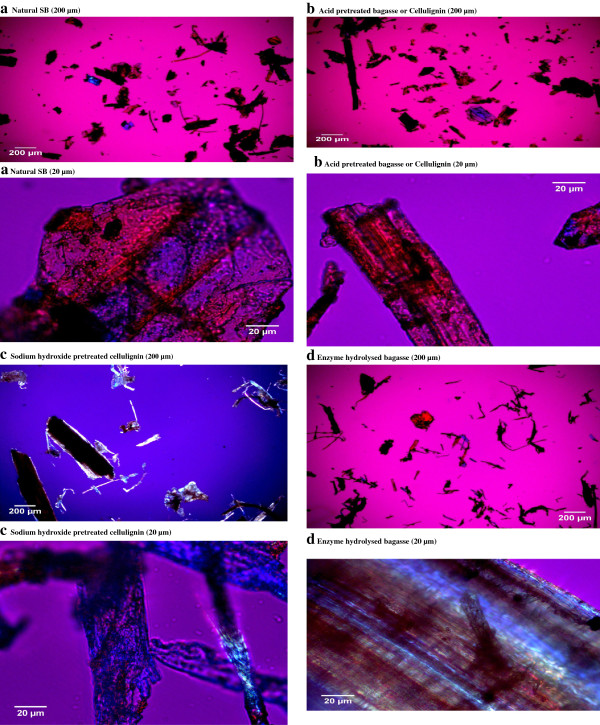
**Transmitted light microscopic (TLM) view of natural sugarcane bagasse (SB), cellulignin (after dilute acid hydrolysis), NaOH pretreatment of cellulignin, and enzyme digested bagasse. (a)** Natural SB (200 and 20 μm), **(b)** acid pretreated bagasse or cellulignin (200 and 20 μm), **(c) s**odium hydroxide pretreated cellulignin (200 and 20 μm), and **(d)** enzyme hydrolyzed bagasse (200 and 20 μm). SB, sugarcane bagasse; TLM, transmitted light microscopy.

TLM images of natural SB and dilute acid pretreated SB (cellulignin), alkali pretreated cellulignin, and enzyme digested substrate are shown in Figure 2. The images of each sample were captured using two magnifications (20 and 200 μm). TLM images presented significant surface changes in the cell wall of pretreated and enzyme digested samples as compared to the native SB. TLM is based on the set-up where the light is transmitted from a source on the opposite side of the sample from the objective. The light on the specimen is passed through via a condenser to gain a very high range of illumination. The image of illuminated bagasse samples appears through the objective lens to oculars presenting an enlarged view. However, there is no clear information available in literature explaining the TLM analysis of pretreated biomass samples. Natural SB showed loose particles of 200 μm size which were almost oval or irregular shaped (20 μm). The natural SB samples were loosely aggregated with brownish and mixed brownish and blue color. Dilute sulfuric acid pretreated samples showed a thin shape of particles (200 μm size) and a more cylindrical shape (20 μm size). Lignin was re-localized on the surface and fibers were very thin. Acid and sodium hydroxide pretreated samples appeared as large aggregates of fibrous tissue. These aggregates were clumped with a deformed shape. The appearance of bagasse particles were rod-shaped with sharp edges corresponding to the different individual cells and fragments thereof which primarily constitute the structure of SB. Similar observations were reported by Shogren *et al*. [24] who analyzed the cell wall surface of acid/alkali pretreated corn stover by polarized light microscopy. Dilute sodium hydroxide pretreated bagasse were less brownish and bluish in color with altered structure revealing lignin removal. The fibers were clumped with decreased lignin content. This appearance of acid–base pretreated SB is more like cellulose (blue appearance). FitzPatrick [25] observed a similar appearance of cellulose (native) and after dissolution with ionic liquids. The enzyme digested samples were highly clumped, with thinned fibers, and were widely spread. The morphology was deformed and irregular showing the removal of the integrated part of the bagasse cell wall.

### X-ray diffraction (XRD)

**Figure 3:**
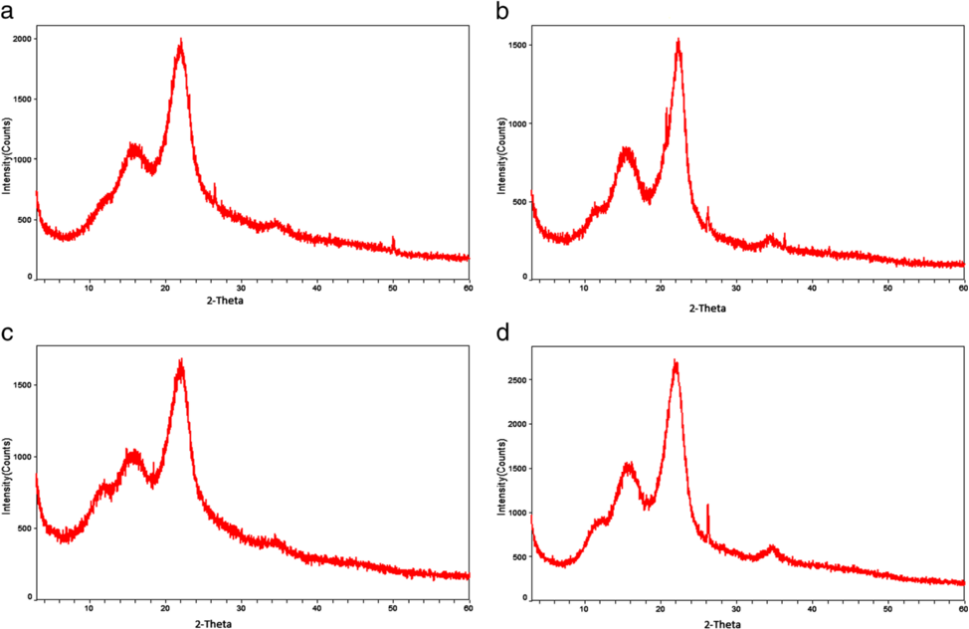
**X-ray diffraction (XRD) pattern of native, cellulignin, sodium hydroxide pretreated, and enzyme hydrolyzed sugarcane bagasse (SB).** The crystallinity index (CrI) was found to be increased in cellulignin and NaOH pretreated bagasse. Enzymatic hydrolyzed SB showed the CrI value of cellulignin and NaOH treated bagasse. **(a)** Natural bagasse, **(b)** dilute sulfuric acid pretreated bagasse (cellulignin), **(c)** dilute sodium hydroxide pretreated cellulignin, and **(d)** enzyme hydrolyzed bagasse. CrI, crystallinity index; SB, sugarcane bagasse; XRD, X-ray diffraction.

The X-ray diffraction (XRD) profile of native, dilute acid pretreated bagasse (cellulignin), sodium hydroxide pretreated cellulignin, and enzyme digested cellulosic substrate is shown in Figure 3a,b,c,d. The crystallinity index (CrI) of all three samples was calculated following the method of Segal *et al*. [26] and Park *et al*. [27]. The intensities (I002) of the amorphous cellulose peak and crystalline cellulose peak were considered to calculate the CrI of all four samples of bagasse. The CrI of native SB was 48.8%. The CrI of acid and alkali pretreated SB was comparatively higher than natural SB showing hikes of CrI due to the sequential increment in cellulose content in these samples (Figure 3b,c). Dilute sulfuric acid pretreatment of bagasse (100 mg of acid/g of dry matter, 121°C, during 20 minutes) removed the hemicellulose, and thus increased the cellulose amount in samples eventually and showed higher CrI (58.82%). Further, cellulignin when pretreated with dilute alkali pretreatment showed maximum CrI (71.87%) because of the removal of lignin, and thus increased the cellulose concentration in bagasse than that of native SB and cellulignin.

Earlier, Rezende *et al*. [7] observed the CrI of raw bagasse (48.7 ± 2.5%), corresponding to a cellulose amount of 35.2%. A liner increment in sample crystallinity was observed with the cellulose amount as the sample was treated with 1% H_2_SO_4_, 0.25%, or 0.5% NaOH, corresponding to cellulose percentages of 51%, 66%, and 68%, respectively. Sindhu *et al*. [28] reported the increased CrI (67.83%) of dilute sulfuric acid plus formic acid pretreated SB samples. Velmurugan and Muthukumar [29] also observed the CrI (66%) of sodium hydroxide pretreated bagasse which was further increased (up to 70.7%) after sono-assisted pretreatment as compared to native SB (50%). The CrI of enzyme digested samples (66.98%) was not found to be increased. Furthermore, additional peaks were more visible in the spectra of enzyme digested bagasse due to the presence of SiO_2_ in enzyme hydrolyzed bagasse samples. This is an intriguing finding, probably due to the high beta-glucosidase concentration in the commercial enzymatic preparations and complexity of the SB cellulose chemical composition and morphology. Earlier, Binod *et al*. [30] reported the CrI of native SB (53.44%), microwave-alkali pretreatment (65.29%), and enzyme hydrolyzed microwave-alkali pretreated bagasse (58.58%).

### Fourier transform infrared (FTIR) spectroscopy

**Figure 4:**
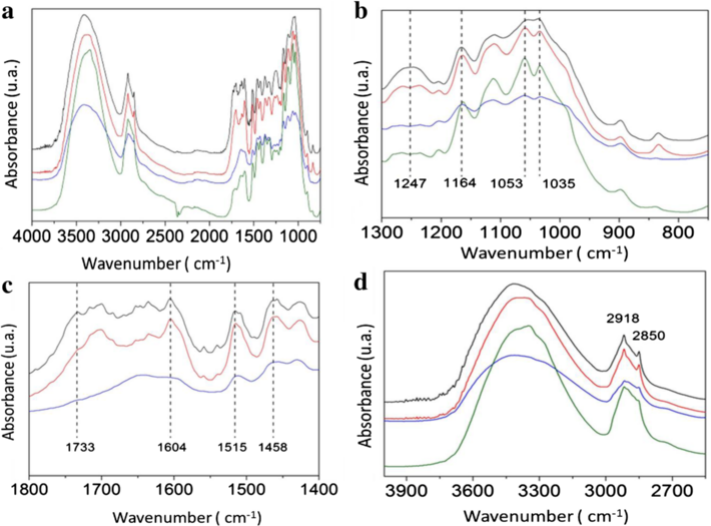
**Fourier transform infrared (FTIR) spectra of sugarcane bagasse (SB). (a)** FTIR spectra of the crushed SB in the range of 4,000 to 1,000 cm^-1^. The black line represents the SB. The red line, blue line, and green line represent the hydrolysis with sulfuric acid (H_2_SO_4_), alkaline hydrolysis with sodium hydroxide (NaOH), and enzymatic hydrolysis, respectively. **(b)** FTIR spectra of the crushed SB in the range of 1,300 to 750 cm^-1^. The black line represents the SB. The red line, blue line, and green line represent the hydrolysis with sulfuric acid (H_2_SO_4_), alkaline hydrolysis with sodium hydroxide (NaOH), and enzymatic hydrolysis, respectively. The vertical dashed line denotes the wave number related to SB, which provides a better comparison. **(c)** FTIR spectra of the crushed SB in the range of 1,800 to 1,400 cm^-1^. The black line represents the SB. The red and blue line represent the acid hydrolysis with sulfuric acid (H_2_SO_4_) and alkaline hydrolysis with sodium hydroxide (NaOH), respectively. The vertical dashed line denotes the wave number related to SB, which provides a better comparison. **(d)** FTIR spectra of the SB in the range of 3,900 to 2,700 cm^-1^. The black line represents the SB. The red line, blue line, and green line are the acid hydrolysis with sulfuric acid (H_2_SO_4_), alkaline hydrolysis with sodium hydroxide (NaOH), and enzymatic hydrolysis, respectively. The vertical dashed line denotes the wave number related to SB, which provides a better comparison. FTIR, Fourier transform infrared; SB, sugarcane bagasse.

The infrared spectra were used to determine changes in the structure of cellulose, hemicellulose, and lignin during the sequence of treatments subjected to bagasse (native), pretreated, and enzymatically hydrolyzed. Figure 4a shows the FTIR spectrum of natural SB and sequential acid–base pretreated followed by enzyme hydrolysis. The band at 898 cm^-1^ is characteristic of the glycosidic bond β-(1 → 4) cellulose [31,32]. The region between 1,200 and 1,100 cm^-1^ is a large contribution of hemicellulose and cellulose, which exhibits a maximum value around 1,035 cm^-1^ due to C-O stretching and 1,164 cm^-1^ for the asymmetrical stretching of C-O-C [33-35]. The region around 1,247 cm^-1^ was due to the stretching of C-O, which is characteristic of hemicellulose and lignin [31].

The region between the wavelengths of 1,450 to 1,300 cm^-1^ was omitted. According to literature, this region exhibits a high molecular coupling, which makes the area quite complex, involving a superposition of several modes of vibration of the lignin and carbohydrates [36]. A band around 1,458 cm^-1^ is reported to be a deformation of lignin CH2 and CH3, and 1,604 cm^-1^ is reported to be stretching of the C = C and C = O lignin aromatic ring. The band around 1,515 cm^-1^ is because of the C = C stretching of the aromatic ring in lignin [33,34]. A band around 1,733 cm^-1^ is characteristic of C = O stretching of unconjugated hemicellulose. The peak around 2,850 cm^-1^ is reported due to the symmetric stretch of CH and CH2, while the peak at 2,918 cm^-1^ is due to asymmetrical stretching of CH2 and CH. Both denote the characteristics of cellulose [36]. The region between 3,800 and 3,000 cm^-1^ covers the related crystalline structure of cellulose. This region represents the sum of the vibration of valence bands of the hydrogen bond of the OH group and the bands of intra-molecular and intermolecular hydrogen bonds [37].

Figure 4b shows the FTIR spectrum of the region 1,300 to 750 cm^-1^ of native SB, sulfuric acid pretreated, NaOH-pretreated cellulignin, and enzymatic digested SB. The region between 1,100 and 1,000 cm^-1^ clearly shows two peaks after acid hydrolysis (red line), indicating the removal of hemicellulose. The removal of hemicellulose is also evident in the region of 1,247 cm^1^. Alkaline hydrolysis (blue line) seems to be affected due to the removal of lignin moieties. After the enzymatic hydrolysis (green line), the peaks between 1,200 and 1,000 cm^-1^ are accentuated, which demonstrates the hydrolysis of cellulose.

Figure 4c shows the FTIR spectrum of the region 1,800 to 1,400 cm^-1^ of native SB, cellulignin, alkaline hydrolyzed cellulignin, and enzymatic digested material. The region of 1,733 cm^-1^ is affected after acid hydrolysis (red line), which indicates the decrease in hemicellulose content. The regions 1,604 cm^-1^, 1,515 cm^-1^, and 1,458 cm^-1^ relating to the lignin macromolecule have noticeable changes after alkaline hydrolysis (blue line). These changes are good evidence of effective lignin degradation after the alkali mediated delignification process.

Figure 4d shows the FTIR spectrum of the region 3,900 to 2,700 cm^-1^ of native SB, acid hydrolyzed bagasse, alkaline hydrolyzed cellulignin, and enzyme hydrolyzed substrate. Bands around 2,918 cm^-1^ and 2,850 cm^-1^ do not seem to change during acid hydrolysis (red line) which is related to the cellulose. This indicates that the major effect of treatment with dilute acid is the removal of hemicellulose. However, this region is affected during alkaline hydrolysis (blue line) and enzymatic hydrolysis (green line). The changes in the two local maxima of 2,918 cm^-1^ and 2,850 cm^-1^ indicate that chemical treatment has also affected the cellulose chain. The increase in line width and asymmetry of the curves in the range of 3,800 to 3,000 cm^-1^ in the course of the treatments indicates disturbances in the crystalline structure of cellulose. These changes are strong evidence of intra-molecular hydrogen bonding disruption in cellulose [38].

### Fourier transform near-infrared (FT-NIR) spectroscopy

**Figure 5:**
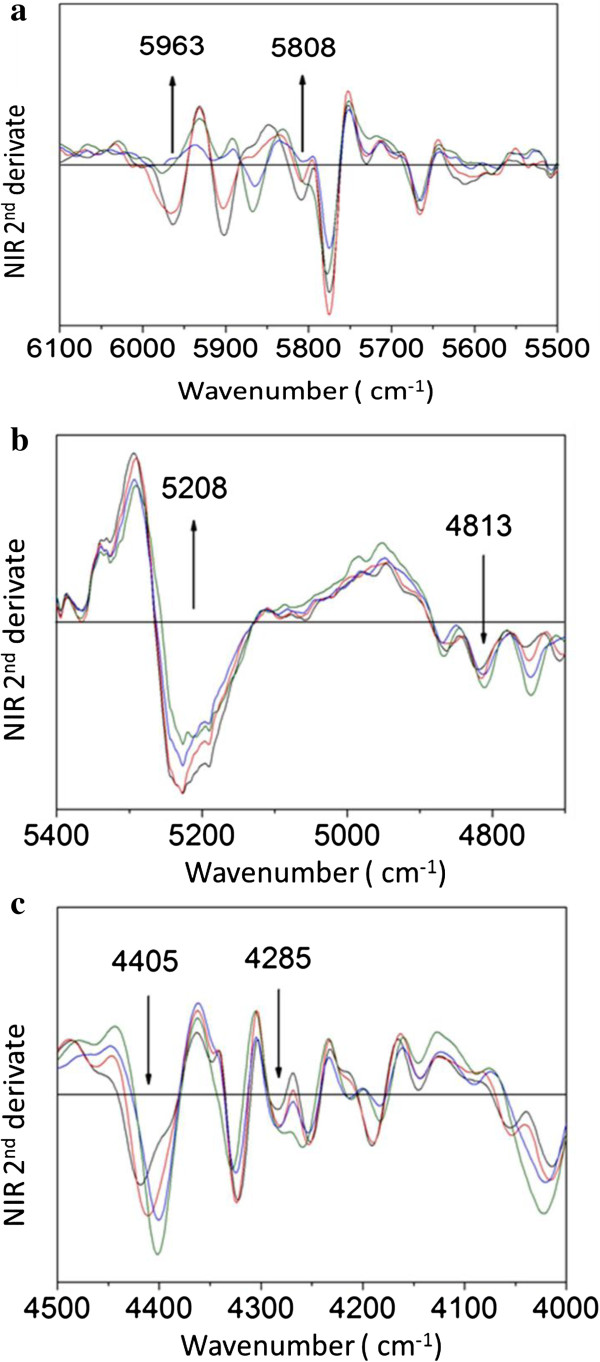
**Second derivative of the absorption spectra of Fourier transform near-infrared (FT-NIR) spectroscopy of sugarcane bagasse (SB). (a)** Second derivative of the absorption spectra of FT-NIR SB in the range of 6,100 to 5,500 cm^-1^. The black line represents the SB. The red line, blue line, and green line is the acid hydrolysis with sulfuric acid (H_2_SO_4_), alkaline hydrolysis with sodium hydroxide (NaOH), and enzymatic hydrolysis, respectively. The arrows denote the vertical wavenumber for the SB, which provides a better view. **(b)** Second derivative of the absorption spectra of FT-NIR SB in the range of 5,500 to 4,700 cm^-1^. The black line represents the SB. The red line, blue line, and green line are the hydrolysis with sulfuric acid (H_2_SO_4_), alkaline hydrolysis with sodium hydroxide (NaOH), and enzymatic hydrolysis, respectively. The arrows denote the vertical wavenumber for the SB, which provides a better view. **(c)** Second derivative of the absorption spectra of FT-NIR SB in the range of 4,500 to 4,000 cm^-1^. The black line represents the SB. The red line, blue line, and green line are the acid hydrolysis with sulfuric acid (H_2_SO_4_), alkaline hydrolysis with sodium hydroxide (NaOH), and enzymatic hydrolysis, respectively. The arrows denote the vertical wavenumber for the SB, which provides a better view. FT-NIR, Fourier transform near-infrared; SB, sugarcane bagasse.

The use of FT-NIR spectroscopy is a method that has been used for the investigation of organic molecules due to its simplicity and speed. Generally, the spectra in the near-infrared bands are the result of harmonics (overtone) containing basic groups, for example CH, OH, and NH [39]. Figure 5a shows the second derivative of the absorption spectrum for the FT-NIR ranging from 6,100 to 5,500 cm^-1^ for native SB, dilute acid pretreated, alkaline hydrolyzed, and enzymatic digested samples. The assignments of polysaccharides and lignin present in SB (native) and pretreated SB samples were considered according to Krongtaew *et al*. [40] who observed a similar spectrum from wood fibers.

Regarding structural changes in hemicellulose after sulfuric acid pretreatment, the region around 5,808 cm^-1^ is the first of the CH stretching band harmonic (overtone CH stretch) attributed to variations of hemicellulose [40,41]. The minimum local amplitude variation in this region indicates the removal of hemicellulose content [40]. The range of the 6,000 to 5,920 cm^-1^ band is attributed to the first harmonic of the stretching vibration of aromatic CH (CH stretching vibration of aromatics) and is responsible for variation of lignin content. The change in local minimum amplitude in this region indicates degradation in units of lignin macromolecules. Figure 5b,c shows the second derivative of the absorption spectra for the FT-NIR range from 5,500 to 4,000 cm^-1^ of native, dilute sulfuric acid pretreated, sodium hydroxide pretreated, and enzyme digested SB. The regions around 4,813, 4,285, and 5,208 cm^-1^ are combinations of the first harmonic of the CH stretching, and the region around 4,405 cm^-1^ is the combination of the first harmonic of CO stretching in polysaccharides [40,41]. According to Figure 5b,c, it is possible to observe changes in the content of polysaccharides. The steps in which the samples are subjected to hydrolysis result in a relative increase in local minimum amplitude, indicating the increase of cellulose in acid pretreated and delignified samples.

### Raman spectroscopy

**Figure 6:**
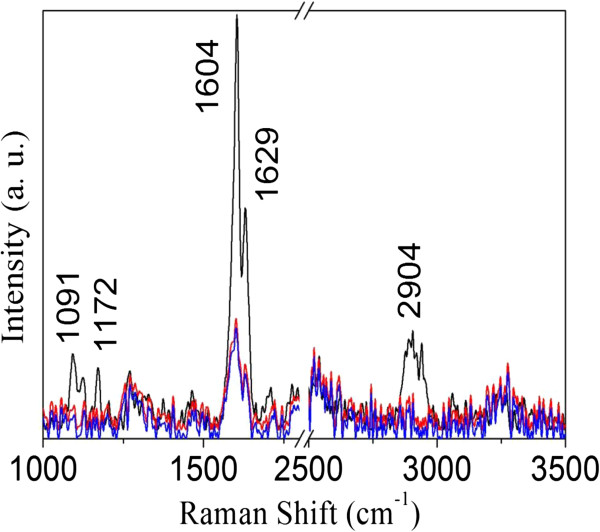
**Raman spectra of sugarcane bagasse (SB).** Raman spectra of native SB (black line), pretreated bagasse with dilute sulfuric acid (red line), and dilute sodium hydroxide pretreated bagasse (blue line). SB, sugarcane bagasse.

The Raman spectra of the native SB, cellulignin, and delignified bagasse are shown in Figure 6. Raman spectra do not clearly show the contribution of hemicellulose in the SB. Hemicellulose has a weak Raman signal with spectral features superposed with the signals of the cellulose and lignin [42]. It is known that the ratio of the bands at 1,172 and 1,204 cm^-1^ reflects the orientation of the cellulose relative to the electric field polarization of the laser light [43]. As a result, our data suggest that the sugarcane fibers are almost aligned with the electric field direction that comes out from the microscope. In Figure 6, the peaks around 1,091 and 2,904 cm^-1^ are attributed to cellulose and hemicellulose, respectively. From the figure, it is evident that the sulfuric acid pretreatment strongly affected the cellulose/hemicellulose bands [10]. On the other hand, the strong band lines at 1,603 and 1,629 cm^-1^ are the result of phenyl groups in lignin [42]. When the acid pretreated SB was delignified, the intensity of this band decreased significantly. Nevertheless, they do not disappear which indicates that some lignin moieties still remain after pretreatment [10,42]. Comparing the intensities, Raman spectroscopy shows a clear reduction in intensity of peaks in acid and alkaline hydrolyzed samples as compared to the native bagasse due to molecular disarrangement during pretreatment. We did not observe any change in Raman spectra of the enzymatically hydrolyzed material.

### Solid-state ^13^C nuclear magnetic resonance (NMR) spectroscopy

**Figure 7:**
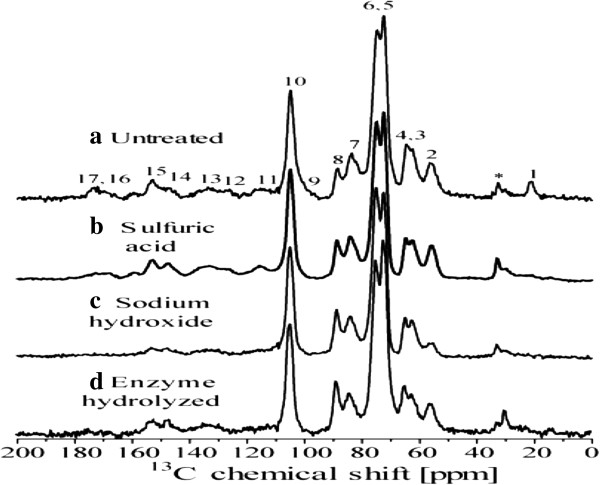
**Solid state**^**13**^**C CPMAS-TOSS nuclear magnetic resonance (NMR) spectra of sugarcane bagasse (SB). (a)** Natural, **(b)** sulfuric acid pretreated, **(c)** sodium hydroxide pretreated, and **(d)** enzyme hydrolyzed SB. The numbers concern the assignments of the signals to specific chemical groups given in Rezende *et al*. [7]. CPMAS-TOSS, cross polarization under magic angle spinning and total suppression of spinning sidebands; NMR, nuclear magnetic resonance; SB, sugarcane bagasse.

Figure 7 shows cross polarization under magic angle spinning and total suppression of spinning sidebands (CPMAS-TOSS) spectra of the solid fractions of untreated and pretreated SB samples. All spectra were normalized with respect to line 10 (C1 carbon of cellulose). The chemical shift assignments were based on the comparison with the ^13^C NMR spectra from wood samples [44,45] and SB with similar pretreatment, as shown in Table 2 and Rezende *et al*. [7]. For the untreated SB sample, the signals in the 50 to 120 ppm region are mainly due to cellulose carbons with smaller contributions from lignin and hemicelluloses. Signals 3 at 63 ppm and 7 at 84 ppm are assigned, respectively, to C6 and C4 carbon from amorphous cellulose [7,46,47], while signals 4 at 65 ppm and 8 at 88 ppm are assigned to C6 and C4 carbon in crystalline cellulose [7,46,47]. Although lignin signals appear in all spectral regions, signals labeled as 2, 11, 12, 13, 14, and 15 in Figure 7a are majorly due to lignin [7], while that marked by * is due to aliphatic lignin carbons not bound to oxygen. Hemicellulose carbons contribute to lines 1, 3, 6, 7, 9, and 17 [7,46,47].

**Table 2:** **Kinetic parameters for ethanol production from detoxified sulfuric acid hydrolysate after detoxification and enzymatic hydrolysates by *Scheffersomyces shehatae *** **UFMG HM 52.2 and *Saccharomyces cerevisiae *** **174, respectively**

**Parameters**	**Hemicellulose hydrolysate**	**Enzyme hydrolysate**
	***S. shehatae***^**a**^	***S. cerevisiae***^**b**^
Initial sugars (g_s_/l)	23.64	36.65
Sugar consumed (%)	100	100
Ethanol produced (g_p_/l)	9.11	8.13
Ethanol yield (g_p_/g_s_)	0.38	0.22
Ethanol productivity (g_p_/l/h)	0.189	0.11
Biomass produced (g_x_/l)	3.34	6.55
Biomass yield (g_x_/g_s_)	0.07	0.178
Biomass productivity (g_x_/l/h)	0.141	0.090

^a^Fermentation of hemicellulose hydrolysate by *S. shehatae* showed maximum ethanol production (9.11 g/l) after 48 hours, thus ethanol productivity was calculated taking 48 hours into consideration. After 48 hours, sugar consumption was 100%. Biomass productivity was also calculated taking 48 hours into consideration. ^b^Fermentation of enzymatic hydrolysate by *S. cerevisiae* showed maximum ethanol production (8.13 g/l) after 72 hours, thus ethanol productivity was calculated taking 72 hours into consideration. After 48 hours, sugar consumption was 100%. Biomass productivity was also calculated taking 72 hours into consideration as it was continuously increasing until 72 hours of incubation.

The spectrum of the sulfuric acid treated sample is shown in Figure 7b. The intensity decrease of signals 1 and 17 as well as the improved spectral resolution in the 50 to 120 ppm region indicates the almost complete removal of hemicellulose in this sample. The spectrum of the samples pretreated by sodium hydroxide is shown in Figure 7c. The intensity decrease of signals *, 2, 11, 12, 13, 14, and 15 show the reduction of the lignin to cellulose fraction in the bagasse sample after the sodium hydroxide treatment. Moreover, in agreement with the Raman results, the remaining lignin signals in the spectra of Figure 7c show that lignin is not completely removed by these treatments.

Figure 7d shows the spectrum of the solid fraction obtained after enzyme hydrolyze of the sodium hydroxide and sulfuric acid treated sample. Assuming that the relative amount of lignin is maintained during the enzyme hydrolysis, the increase of lignin signals, lines *, 2, 11, 12, 13, 14, and 15, relative to cellulose ones, lines 3, 4, 5, 6, 7, 8, and 10, is associated to the removal of cellulose. Information that can be obtained from Figure 7 is the increase in the crystallinity of the cellulose after the enzymatic treatment. As already mentioned, signals at 84 and 88 ppm are due to the amorphous and crystalline cellulose, respectively. Spectra of Figure 7c,d show the change in the relative intensity of signal 7 and 8 as well as 3 and 4, which might suggests an increase in crystallinity of cellulose upon enzymatic hydrolysis, showing the preference for removal of amorphous cellulose. However, it is worth pointing out that there is indeed a lignin peak in the region of the C4 signal which may compromise the calculation of the ratio between the crystalline and amorphous signal in the spectrum of the enzymatic treated sample. Zhao *et al*. [48] observed that the ordered structure of crystalline cellulose was not found to be disrupted after the hydrolysis of cellulose. They found that the peak ratio of C4 (79 to 86 ppm)/C4 (86 to 92 ppm) to calculate the cellulose crystallinity remains the same after the hydrolysis reaction. The relative ratio of amorphous cellulose and crystalline cellulose was almost similar prior to hydrolysis.

### Ethanol fermentation

#### *Fermentation of acid hydrolysate by* Scheffersomyces shehatae

**Figure 8:**
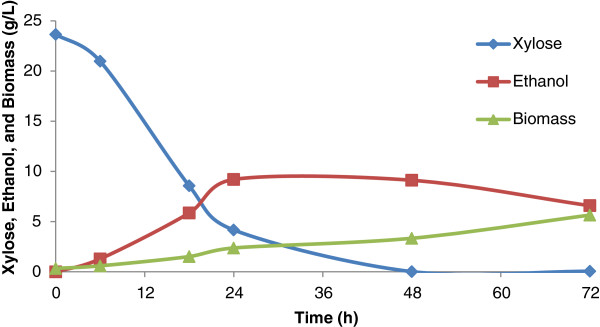
**Time course of growth, sugar utilization, and ethanol production using detoxified sulfuric acid hydrolysate (fermentable pH of the hydrolysate was adjusted 5.5) by *Scheffersomyces shehatae *** **UFMG HM 52.2 at 30°C.**

A typical fermentation profile of detoxified SB acid hydrolysate using *S. shehatae* UFMG HM 52.2 is shown in Figure 8. The yeast *S. shehatae* UFMG HM 52.2 revealed maximum ethanol production (9.11 g/l) with the yield (0.38 g/g) and productivity (0.19 g/l/h) from detoxified hemicellulosic acid hydrolysate after 48 hours, and, thereafter, it declined (Figure 8). However, biomass production (5.64 g/l) continued to increase up to the completion of the fermentation cycles (72 hours). Table 2 shows the kinetic parameters of fermentation of detoxified hemicellulosic hydrolysate into ethanol. *S. shehatae* UFMG HM 52.2 is a native xylose fermenting yeast which was isolated from a rotting wood sample in an Atlantic rainforest site of Brazil. Earlier, this strain was employed for the fermentation of oxalic acid pretreated hemicellulosic hydrolysate, which showed ethanol production (3.20 g/l) with a yield of 0.35 g/g and productivity of 0.13 g/l/h after 24 hours, followed by a declination [10]. Biomass (0.38 g/g) was found to be continuously increasing until the completion of the fermentation cycle with the productivity of 0.049 g/l/h. Chandel *et al*. [19] also reported a regular increase in biomass of *S. shehatae* NCIM 3501 even after the exhaustion of xylose in 24 hours with the utilization of ethanol as a carbon source for the metabolic growth. Sánchez *et al*. [49] found ethanol production (4.5 g/l) from *S. shehatae* CBS 4410 growing on *Paja brava* acid hydrolysate (19.8 g/l xylose and 2.5 g/l glucose). Sreenath and Jeffries [50] reported 34 g/l of ethanol with a yield of 0.46 g/g from *S. shehatae* FPL-Y-049 growing on wood hemicellulose hydrolysate.

#### *Fermentation of enzyme hydrolysate by* Saccharomyces cerevisiae

**Figure 9:**
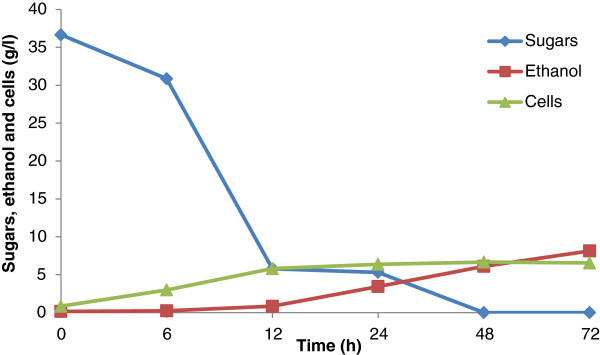
**Time course of growth, sugar utilization, and ethanol production using enzymatic hydrolysate (pH of the hydrolysate 5.5) by *Saccharomyces cerevisiae *** **174 at 30°C.**

Figure 9 shows the fermentation profile of *S. cerevisiae* 174 utilizing enzymatic hydrolysate, which contains only glucose as a carbon source (36.60 g/l). Enzymatic hydrolysates were devoid of any fermentation inhibitors. When fermented with *S. cerevisiae* using enzymatic hydrolysate, the maximum ethanol production (8.13 g/l) was found with the yield (0.22 g/g) and productivity (0.11 g/l/h) after 72 hours (Table 2). After 72 hours of fermentation, maximum biomass production (6.55 g/l) was obtained with the yield (0.17 g/g) and productivity (0.09 g/l/h). This strain showed higher biomass production rates than *S. shehatae* UFMG HM 52.2 under the employed fermentation conditions. Previously, *S. cerevisiae* 174 showed maximum ethanol production (6.6 g/l, yield 0.46 g/g) from enzymatic hydrolysate (18.4 g/l glucose) of oxalic acid pretreated SB [10]. Given the cultivation conditions in the present study, this strain favored higher biomass production than ethanol. Martin *et al*. [16] observed maximum ethanol production (7.4 g/l, yield 0.28 g/g, and productivity 0.37 g/l/h) from *S. cerevisiae* ATCC 96581 grown on detoxified SB hydrolysate (26.0 g/l total sugars). Earlier, Chandel *et al*. [51] observed ethanol production 19.45 ± 0.55 g/l (yield 0.41 ± 0.01 g/g) from natural *S. cerevisiae* VS_3_ using enzymatic hydrolysate of *Saccharum spontaneum* (53.91 ± 0.44 g/l TRS).

## Conclusions

Plant cell walls are a useful source of renewable energy. For the biochemical ethanol production from lignocellulosics, it is essential to overcome the complex, rigid, and recalcitrant characteristics of the plant cell wall. Chemical pretreatment encompassing sequential acid–base pretreatment of SB separates hemicellulose and lignin, and increases the accessibility of cellulose to cellulase enzyme mediated action to convert into glucose. The microscopic (SEM and TLM) and spectroscopic techniques (FTIR, FT-NIR, Raman, NMR, and XRD) used in this work provided in-depth structural investigation of chemical changes at the molecular level during sequential pretreatment and enzymatic digestion.

Dilute sulfuric acid pretreatment significantly removed hemicellulose (10.9 g/l xylose) in addition to lignin relocalization. Cellulignin was further delignified by dilute sodium hydroxide pretreatment, which efficiently removed lignin from the substrate eventually increasing the cellulose fraction in the substrate. Acid–base pretreated substrate showed efficient enzymatic action toward the depolymerization of cellulose into glucose (0.91 g sugars/g pretreated bagasse). Detoxified hemicellulosic hydrolysate, when fermented by *S. shehatae* UFMG HM 52.2, showed ethanol production of 9.11 g/l (yield 0.38 g/g). The cellulosic hydrolysate showed ethanol production of 8.13 g/l (yield 0.22 g/g) by *S. cerevisiae* 174. Both microorganisms showed a moderate ethanol yield which needs further investigation. Both microorganisms are native and showed average ethanol production potential from SB hydrolysates. System metabolic engineering-based approaches, improvements in media formulation, and modified fermentation methods could provide the desired ethanol yields from these microorganisms growing on lignocellulose hydrolysates.

## Material and methods

### Preparation of raw substrate

The raw substrate, SB, was acquired from Usina Vale do Rosário (Morro Agudo, São Paulo, Brazil). During preliminary processing, the SB was air-dried and knife-milled (model number MA 680; Marconi Equipamentos, Piracicaba, São Paulo, Brazil) to pass through with a 20-mesh sieve. The finely milled SB was washed under running tap water to remove the dust and dried at 45°C for further experiments.

### Dilute acid hydrolysis

The dilute acid hydrolysis of SB was carried out in a Parr reactor 4848 (Moline, IL, USA) with a capacity of 5 l. For the hydrolysis of the SB, H_2_SO_4_ (98% purity) was used as a catalyst in a ratio of 100 mg of acid/g of SB, at 121°C for 20 minutes, using a ratio of 1/10 between the bagasse mass and the volume of acid solution. After the reaction, the solid material (cellulignin) was recovered by filtration using muslin cloth. The hydrolysate obtained was maintained at 4°C. Cellulignin was washed with running tap water until neutral pH and dried at 45°C.

### Detoxification of SB hemicellulosic hydrolysate

The SB hemicellulosic hydrolysate was vacuum concentrated at 70°C in a concentrator and further detoxified by sequential calcium oxide-activated charcoal pretreatment according to Alves *et al*. [52]. The hydrolysate was finally filtered under vacuum and then autoclaved under 0.5 atm (110°C) for 15 minutes.

### Dilute sodium hydroxide pretreatment

Cellulignin obtained after dilute acid hydrolysis was subsequently pretreated by sodium hydroxide mediated delignification. It was carried out in a Parr reactor 4848 of 5 l capacity. Sodium hydroxide (1% m/v) was used as a catalyst in a ratio of 1/10 between the cellulignin mass and the volume of alkali solution, at 121°C for 1 hour. After the reaction, the solid material (cellulose) was recovered by filtration using muslin cloth. The recovered solid residue was washed with running tap water until neutral pH and dried at 45°C.

### Enzymatic hydrolysis

Enzymatic hydrolysis of acid–base pretreated bagasse was performed in a 250 ml Erlenmeyer flask containing 7.5 g d.wt. of acid–base pretreated bagasse and 100 ml of citrate buffer (50 mM, pH 4.8). Substrates with buffer were pre-incubated at room temperature for 90 minutes. The substrate soaked in citrate buffer was supplemented with cellulase loadings (15 FPU/g of the dry substrate from Celluclast 1.5 L and 20 IU/g of β-glucosidase from Novozym 188). Surfactant (Tween 20) was also added (0.10 g/g substrate) in the hydrolysis experiment. Enzymatic hydrolysis was performed at 50°C at 150 rpm in an incubator shaker (Innova 4000; New Brunswick Scientific, Enfield, CT, USA). The enzymes were purchased from Sigma Aldrich (St Louis, MO, USA). The enzymatic hydrolysis was performed for a period of time up to 96 hours. Samples were collected after every 24 hours, centrifuged, and analyzed to determine the sugars released.

### Analysis

The chemical composition of the solid material (raw bagasse, cellulignin, and cellulosic pulp (1% m/v NaOH, 120°C, 90 minutes)) was analyzed by a methodology validated by Gouveia *et al.*[53]. The determination of constituent concentrations in dilute acid hydrolysate and enzymatic hydrolysates were verified by HPLC. The content of glucose, xylose, arabinose, formic acid, and acetic acid were verified in chromatograph Shimadzu LC-10AD (Kyoto, Japan) with a column equipped with Aminex HPX-87H (300 × 7.8 mm; Bio-Rad, Hercules, CA, USA), coupled to a refractive index detector (RID-6A), and 0.01 N sulfuric acid as an eluent at a flow rate of 0.6 ml/min, column temperature of 45°C, and injected volume of 20 μl. The samples were previously filtered through a Sep-Pak C18 filter (Sigma Aldrich, USA). The determination of furfural and HMF was obtained in chromatograph Shimadzu LC 10AD with column HP-RP18 (200 × 4.6 mm), coupled to an ultraviolet detector SPD-10A UV–VIS in a wavelength of 276 nm, with eluent acetonitrile/water (1/8), and 1% of acetic acid. The used flow was 0.8 ml/min, the column temperature was 25°C, and the volume injected was 20 μl. All the samples were filtered in membrane Minisart 0.22 μm (Sartorius, Epsom, UK) before the readings. TRS in enzyme hydrolysates were estimated by using spectrophotometer (Beckman DU-640B; Beckman Coulter, Brea, CA, USA) following the dinitrosalicylic acid (DNS) method of Miller [54]. All the experiments were carried out in triplicates. The values are the mean of three replicates.

### Ethanol fermentation

*S. shehatae* UFMG HM 52.2 was isolated from a sample of rotting wood grown in xylan medium (1% xylan, 0.67% yeast nitrogen base, 0.02% chloramphenicol, and pH 5.0 ± 0.2), collected in a private natural reserve, Bello and Kerida, located in Rio Grande de Cima, Nova Friburgo, Rio de Janeiro, Brazil. The strains were maintained on yeast extract peptone dextrose (YPD) plates and stored at 4°C. *S. shehatae* UFMG HM 52.2 was grown in 150 ml Erlenmeyer flasks containing 50 ml of seed medium (30 g/l of xylose), 20 g/l of peptone, and 10 g/l of yeast extract in an orbital incubator shaker at 30°C, 200 rpm. Synthetic medium consisted of commercial xylose (37 g/l), and the other medium ingredients were the same as the hydrolysate supplemented medium. For the fermentation of *S. shehatae* UFMG HM 52.2, the medium was composed of the hydrolysates supplemented with yeast extract (3.0 g/l), malt extract (3.0 g/l), and ammonium sulfate (5.0 g/l), as described by Parekh *et al*. [55]. Flasks were maintained in a rotator shaker at 30°C and 200 rpm for 72 hours. Samples were collected at 0 hours, 6 hours, 14 hours, 24 hours, and 48 hours to determine the residual sugars and ethanol and biomass production.

*S. cerevisiae* 174 was isolated from the Atibaia River, São Paulo state, Brazil. It was grown in 150 ml Erlenmeyer flasks containing 50 ml of seed medium (30 g/l of glucose, 5 g/l of peptone, 3 g/l of yeast extract, and 0.25 g/l diammonium hydrogen phosphate) in an orbital incubator shaker at 30°C, shaken at 100 rpm. Following 24 hours of growth, fermented broth was centrifuged and *S. cerevisiae* was prepared corresponding to 1.0 g/l cells (d.wt.). Inoculums were aseptically transferred into enzymatic hydrolysates (50 ml) supplemented with medium ingredients.

### Structural analysis

#### Scanning electron microscope (SEM)

The SEM analysis of native, dilute sulfuric acid pretreated, dilute sodium hydroxide pretreated, and enzymatically hydrolyzed SB was performed as described by Kristensen *et al*. [56]. Briefly, native, acid–base pretreated, and enzymatically hydrolyzed SB were distributed on a 12 mm glass coverslip coated with poly-L-lysine (Sigma Diagnostics, São Paulo, Brazil). The dried sections were mounted on aluminum stubs, sputter-coated (JEOL JFC-1600) with a gold layer, and used for scanning. The prepared samples were scanned and imaged using Hitachi S520 SEM (Tokyo, Japan).

### Transmitted light microscopy (TLM)

TLM analysis of native, dilute sulfuric acid pretreated, dilute sodium hydroxide pretreated, and enzyme digested SB samples was performed to reveal the changes in surface morphology in response to transmitted light. After the light passes through the samples, the image of the specimen goes through the objective lens and to the oculars where the enlarged image is viewed. The samples were mounted on aluminum stubs and the light was passed through a condenser to focus it on the samples to obtain very high illumination with the microscope (Axioskop 40; Zeiss, Oberkochen, Germany) and camera (Axiocam ICC 3; Zeiss). All the images were captured through 25, 50, and 400× magnifications.

### X-ray diffraction (XRD)

The crystalline nature of native, dilute sulfuric acid treated, dilute sodium hydroxide pretreated, and enzyme digested SB samples was analyzed by using a Rotaflex diffractometer (model RU200B; Rigaku Tokyo, Japan) using monochromatic CuKa radiation (1.54 Å) set at 40 KV, 30 mA. The goniometer scanned a 2θ range between 5° and 65° at a 2°/min scanning rate. Samples were scanned over the range of 100 <2θ <500 with a step size of 0.05° and the CrI was determined using the empirical method proposed by Segal *et al*. [26] and Park *et al*. [27]. Samples were measured in duplicates and the average values of the CrI was obtained from the relationship between the intensity of the 002 peak for cellulose I (I002) and the minimum dip (Iam) between the 002 and the 101 peaks, following the formula:

$$\mathrm{CrI}=\frac{I\;002-I\;\mathrm{amorphous}}{I\;002}\times 100\%$$

### Fourier transform infrared (FTIR) spectroscopy

FTIR spectroscopic analysis of native, dilute sulfuric acid pretreated, dilute sodium hydroxide pretreated, and enzyme digested SB samples was performed to detect the changes in functional groups. Samples were milled in agate cups for 1.5 hours with 400 rpm (PM 400; RETSCHW, Haan, Germany), followed by passing through a 100-mesh sieve (A Bronzinox, São Paulo, Brazil) with an aperture of 150 μm. The pellets were prepared by mixing 300 mg of spectroscopic grade KBr with 3 mg of sample in an agate mortar. Each sample was then submitted to 10 tons for 3 minutes in a hydraulic press (Atlas 25T; Specac, Swedesboro, NJ, USA). The spectra were collected in the range 4,000 to 400 cm^-1^ with a resolution of 4 cm^-1^ and 72 scans per sample on a VERTEX 70 spectrometer (Bruker Optics, Ettlingen, Germany).

### Fourier transform near-infrared (FT-NIR) spectroscopy

The FT-NIR spectroscopy of native, dilute sulfuric acid pretreated, dilute sodium hydroxide pretreated, and enzyme digested SB samples was performed with a spectrometer FT-NIR multi-purpose analyzer (MPA) from Bruker Optics. The measurements were used to diffuse reflectance, which was analyzed via an integrating macro sample sphere, the diameter of the measured area was 15 mm, and 32 scans per sample were performed with a resolution of 4 cm^–1^ covering a range from 13,000 to 3,500 cm^–1^. Second derivative spectra were calculated with 21 smoothing points after unit vector normalization. All calculations were conducted with OPUS version 6.5 software.

### Raman spectroscopy

The Raman spectra of native and sequential acid–base pretreated SB samples (dilute sulfuric acid pretreatment of bagasse followed by dilute sodium hydroxide pretreatment) were carried out using a Bruker Optics RFS 100 instrument and Nd:YAG laser operating at 1,064 nm. The instrument with 4 cm^-1^ of spectral resolution was equipped with a germanium detector cooled with liquid nitrogen and coupled to a RamanScopeIII microscope system. Good signal-to-noise ratios were obtained with 600 scans, using a range of laser power between 50 and 200 mW.

### Solid-state ^13^C nuclear magnetic resonance (NMR) spectroscopy

Solid-state ^13^C NMR spectroscopy of native, dilute sulfuric acid pretreated, dilute sodium hydroxide pretreated, and enzyme digested SB samples was performed using a Varian Inova spectrometer (Eugene, OR, USA) at ^13^C and ^1^H frequencies of 88.02 and 350.50 MHz, respectively [7]. A Varian 7 mm magic angle spinning (MAS) double resonance probe head was used. Spinning frequencies of 4.5 kHz were controlled by a Varian pneumatic system that ensures a rotation stability of approximately 2 Hz. Radio frequency ramped cross-polarizations under magic angle spinning (CPMAS) combined with total suppression of spinning sidebands (TOSS) and heteronuclear ^1^H decoupling (CPMAS-TOSS) was used to acquire the ^13^C spectra. Typical π/2 pulse lengths of 4.0 μs (^13^C) and 4.5 μs (^1^H), cross-polarization time of 1 ms, acquisition time of 30 ms, and recycle delays of 2 seconds were used in all NMR experiments.

## Abbreviations

CBU: Cellobiase unit; CPMAS-TOSS: Cross polarization under magic angle spinning and total suppression of spinning sidebands; CrI: Crystallinity index; DNS: Dinitrosalicylic acid; FPU: Filter paper unit; FTIR: Fourier transform infrared; FT-NIR: Fourier transform near-infrared; HMF: Hydroxymethylfurfural; HPLC: High performance liquid chromatography; MAS: Magic angle spinning; MPA: Multi-purpose analyzer; Nd:YAG: Neodymium-doped yttrium aluminum garnet; NMR: Nuclear magnetic resonance; RID: Refractive index detector; SB: Sugarcane bagasse; SEM: Scanning electron microscopy; TLM: Transmitted light microscopy; TOSS: Total suppression of spinning sidebands; TRS: Total reducing sugars; XRD: X-ray diffraction; YPD: Yeast extract peptone dextrose.

## Competing interests

The authors declare that they have no competing interests.

## Authors’ contributions

AKC planned and performed the biomass pretreatment, enzymatic hydrolysis, ethanol fermentation, as well as the analysis of the results and manuscript writing. AKC also coordinated the overall study. FAFA assisted in biomass characterization, fermentation experiments, and helped to draft the manuscript. VA, MJVB, and LNR jointly carried out the Raman spectroscopy, FTIR, and FT-NIR analysis, and wrote related text in the manuscript. IP, ODB, and ERA jointly performed the XRD and NMR analysis, and analyzed the results. CAR and FCP provided the yeast strains and fermentation methodology, and both analyzed the fermentation results and contributed to drafting the text related to fermentation. SSS coordinated the overall study, analyzed the results, and finalized the manuscript. All authors suggested modifications to the draft and approved the final manuscript.
